# Investigating the effect of depression on clinical symptoms of temporomandibular disorder in young stressful men

**DOI:** 10.1002/cre2.909

**Published:** 2024-06-16

**Authors:** Maryam Sadrzadeh Afshar, Behzad Salari, Sina Varasteh Nejad

**Affiliations:** ^1^ Department of Oral and Maxillofacial Medicine, Faculty of Dentistry Aja University of Medical Sciences Tehran Iran; ^2^ Department of Orthodontics, Faculty of Dentistry, Tehran Medical Sciences Islamic Azad University Tehran Iran; ^3^ Department of Endodontics, School of Dentistry Aja University of Medical Sciences Tehran Iran

**Keywords:** anxiety, conscripts, depression, stress, temporomandibular disorder

## Abstract

**Objectives:**

The objective of this study was to investigate the relationship of the depression level with temporomandibular disorders (TMDs) in young conscripts as a population with chronic stresses.

**Material and Methods:**

A total number of 144 male conscripts with chronic stress and different levels of depression were assigned to four groups according to the Beck's Depression Inventory (BDI). The control group consisted of age‐matched male conscripts without chronic stress. The diagnosis of TMD was made according to the Diagnostic Criteria for Temporomandibular Disorders (DC/TMD). Data were analyzed using Mann–Whitney and chi‐square tests.

**Results:**

The participants with severe depression were significantly more susceptible to have TMD (*p* = .001) followed by the moderate depression, borderline clinical depression, mild mood disturbance, and control groups. The TMD diagnoses were more prevalent within depression groups compared with the control population (*p* = .01).

**Conclusions:**

The level of depression is directly associated with the presence of TMD in young men with chronic stress.

## 1 INTRODUCTION

Temporomandibular disorder (TMD) is among the most common causes of nonodontogenic pain in the orofacial region. This chronic condition is more prevalent in young women (LeResche, 1997). TMD is not a life‐threatening condition but can considerably affect the quality of life (Bitiniene et al., 2018).

The etiology of TMD is considered multifactorial and influenced by several risk factors, including biological factors, anatomical factors, trauma, occlusal changes, nocturnal movement disorders, and psychosocial factors such as emotions, stress, pain, catastrophizing, and anxiety. The Orofacial Pain Prospective Evaluation and Risk Assessment (OPPERA) assessed several risk factors and evaluated biological, psychosocial, clinical, and health status characteristics (De La Torre Canales et al., 2018; Maixner et al., 2011). Previous data showed that female hormones could affect TMD, but no direct relationship was found between TMD and male hormones (Dalewski et al., 2020).

Some older studies reported a prevalence rate of 7%–14%, while more recent cross‐sectional studies reported higher prevalence rates up to 27% for the adolescents with TMD symptoms (List et al., 1999; Minghelli et al., 2014). It has been estimated that 60%–70% of the general population experiences at least one sign or symptom of TMD in their life time; however, only 5% of individuals show severe TMD that requires treatment (Poveda Roda et al., 2007). Recent studies showed that psychosocial factors such as stress, anxiety, alexithymia, and catastrophizing could affect the onset of TMD and its response to treatment (De la Torre Canales et al., 2020; Rollman & Gillespie, 2000; Sadrzadeh‐Afshar et al., 2023; Shahnavazi et al., 2021). Different malocclusions have also been a controversial risk factor for TMD (Manfredini et al., 2017; Salari et al., 2024). The diagnosis is based on clinical examinations, past medical and dental history, and paraclinical data (Daniele Kapos et al., 2020; Manfredini et al., 2023; Padisar et al., 2020).

Depression is a common psychosocial disorder in modern societies and is recognized as an important cause of global disability (Reddy, 2016). There is a direct but complicate relationship with pain and pain releasing parameters (Fishbain et al., 1997; Linton & Bergbom, 2011). Based on the biopsychosocial theory, somatic health and psychological status were proven tangibly interconnected in TMD patients (Hadjistavropoulos et al., 2011). Previous studies conducted in Poland and Belgium demonstrated that chronic pain due to TMD could deteriorate depression or complicate its treatment process (Simoen et al., 2020; Smardz et al., 2019; Sójka et al., 2019). Moreover, the potential association of TMD with depression and stress has been previously confirmed (Wieckiewicz et al., 2022). A well‐designed cohort study conducted in China reported that depressed individuals had 2.21‐ to 2.64‐fold higher risk of TMD (Liao et al., 2011). Some authors investigated the relationship between psychiatric disorders and facial pain in an Italian population (Mongini et al., 2007). They demonstrated that TMD had a high degree of comorbidity with depression. It was reported that patients with TMD and depression had a poorer response to depression treatment, probably due to increased pain perception threshold (Dickens et al., 2003; Thompson et al., 2016). This depression could arise from many reasons, including jaw trauma and fractures (Salari et al., 2022).

Unlike some previous studies on college students with chronic stress, the present study chose conscripted young men as the target population. The authors believe that male conscripts are more susceptible to chronic stress than college students, and, therefore, this population is more likely to show psychological disorders such as depression. The authors found few well‐designed studies that focused on the relationship of depression and TMD in a population with chronic stress. Therefore, this study was designed to assess the effect of the depression level on clinical symptoms of TMD in young male conscripts.

## 2 MATERIALS AND METHODS

### 2.1 Ethics statement

This cross‐sectional study was conducted in accordance with the Good Clinical Practice, the National Research Council's guide, and the international ethical guidelines, laws, and regulations. It was also approved by the Ethics Committee of Iran (code no: 1398.264). The present study enrolled male conscripts who were willing to participate in the study and signed informed consent forms.

### 2.2 Study participants

A total of 830 Iranian male conscripts were randomly selected from those referred for annual dental examinations to the school of dentistry. We applied one‐stage cluster sampling method for selection of conscripts among the abovementioned population. The study population consisted of 18–25‐year‐old healthy males who were serving their first 6 months of military service. Of 830 subjects, 144 were selected for this study. The young healthy male conscripts with chronic stress and depression who had Angle's class I dental and skeletal relationship were included in this study. Those with noticeable occlusal disharmonies, temporomandibular joint (TMJ) deformities, or history of trauma or orthognathic surgery were excluded. The stress was evaluated according to the Trier Inventory for Chronic Stress (TICS), and levels of depression were assessed according to the Beck's Depression Inventory (BDI). The control group consisted of 138 healthy males aged between 18 and 25 years who were selected among those serving in the army but did not have chronic stress or depression. They were randomly selected among those seeking dental treatment. The same inclusion criteria were also applied to the control group.

### 2.3 Evaluation of stress

The TICS questionnaire was administered among 830 conscripted young males to screen those with chronic stress. It evaluates nine factors, including work and social overload, pressure to perform, excessive demands, lack of social recognition, social tensions, social isolation, and chronic worrying (Table 1).

**Table 1:** English short version of the Trier Inventory for Chronic Stress.

Item	Scale
Arguments I get involved in frequently become lasting conflicts	Social Tensions
I feel that my performance is not recognized enough	Lack of Social Recognition
I feel overwhelmed by my tasks	Work Overload
In spite of the effort I make, I am unable to manage my tasks properly	Excessive Demands from Work
Sometimes I feel overburdened by my responsibilities toward others	Social Overload
I must meet responsibilities which I am adamantly opposed to	Work Discontent
Sometimes I am consumed by my worries	Chronic Worrying
Sometimes I lack the opportunity to articulate my concerns I have no opportunity to discuss things with others	Social Isolation
There are situations in which I find it difficult to be obliging	Pressure to Perform

### 2.4 Evaluation of depression

The BDI was applied for screening and evaluation of the severity of depression among the participants based on 21 items (Table 2). The sum of scores (ranging from 0 to 63) indicated the level of depression of each participant (Beck et al., 1961):

**Table 2:** Beck Depression Inventory.

Symptom	Items
Mood	I do not feel sad/I feel blue or sad/I am blue or sad all the time and I can't snap out of it/I am so sad or unhappy that it is very painful/I am so sad or unhappy that I can't stand it
Pessimism	I am not particularly pessimistic or discouraged about the future // I feel discouraged about the future/I feel I have nothing to look forward to/I feel that I won't ever get over my troubles/I feel that the future is hopeless and that things cannot improve
Sense of failure	I do not feel like a failure/I feel I have failed more than the average person/I feel I have accomplished very little that is worthwhile or that means anything/As I look back on my life, all I can see is a lot of failures/I feel I am a complete failure as a person (parent, husband, wife)
Lack of satisfaction	I am not particularly dissatisfied/I feel bored most of the time/I don't enjoy things the way I used to/I don't get satisfaction out of anything anymore/I am dissatisfied with everything
Guilty feeling	I don't feel particularly guilty/I feel bad or unworthy a good part of the time/I feel quite guilty/I feel bad or unworthy practically all the time now/I feel as though I am very bad or worthless
Sense of punishment	I don't feel I am being punished/I have a feeling that something bad may happen to me/I feel I am being punished or will be punished/I feel I deserve to be punished/I want to be punished
Self‐hate	I don't feel disappointed in myself/I am disappointed in myself/I don't like myself/I am disgusted with myself/I hate myself
Self‐accusations	I don't feel I am any worse than anybody else/I am very critical of myself for my weaknesses or mistakes/I blame myself for everything that goes wrong/I feel I have many bad faults
Self‐punitive wishes	I don't have any thoughts of harming myself/I have thoughts of harming myself but I would not carry them out/I feel I would be better off dead/I have definite plans about committing suicide/I feel my family would be better off if I were dead/I would kill myself if I could
Crying spells	I don't cry any more than usual/I cry more now than I used to/I cry all the time now. I can't stop it/I used to be able to cry but now I can't cry at all even though I want to
Irritability	I am no more irritated now than I ever am/I get annoyed or irritated more easily than I used to/I feel irritated all the time/I don't get irritated at all at the things that used to irritate me
Social withdrawal	I have not lost interest in other people/I am less interested in other people now than I used to be/I have lost most of my interest in other people and have little feelings for them/I have lost all my interest in other people and don't care about them at all
Indecisiveness	I make decisions about as well as ever/I am less sure of myself now and try to put off making decisions/I can't make decisions any more without help/I can't make any decisions at all any more
Body image	I don't feel I look any worse than I used to/I am worried that I am looking old or unattractive/I feel that there are permanent changes in my appearance and they make me look unattractive/I feel that I am ugly or repulsive looking
Work inhibition	I can work about as well as before/It takes extra effort to get started at doing something/ I don't work as well as I used to/I have to push myself very hard to do anything/I can't do any work at all
Sleep disturbance	I wake up more tired in the morning than I used to/I wake up 1‐2 h earlier than usual and find it hard to get back to sleep/I wake up early every day and can't get more than 5 h sleep
Fatigability	I don't get any more tired than usual/I get tired more easily than I used to/I get tired from doing anything/I get too tired to do anything
Loss of appetite	My appetite is no worse than usual/My appetite is not as good as it used to be/My appetite is much worse now/I have no appetite at all any more
Weight loss	I haven't lost much weight, if any, lately/I have lost more than 5 pounds/I have lost more than 10 pounds/I have lost more than 15 pounds
Somatic preoccupation	I am no more concerned about my health than usual/I am concerned about aches and pains or upset stomach or constipation or other unpleasant feelings in my body/I am so concerned with how I feel or what I feel that it's hard to think of much else/I am completely absorbed in what I feel
Loss of libido	I have not noticed any recent change in my interest in sex/I am less interested in sex than I used to be/I am much less interested in sex now/I have lost interest in sex completely

A total score below 10 was considered normal.

A total score between 11 and 16 indicated mild mood disturbance.

A total score between 17 and 20 indicated borderline clinical depression.

A total score between 21 and 30 indicated moderate depression.

A total score between 31 and 40 indicated severe depression.

A total score over 40 indicated extreme depression.

### 2.5 Evaluation of TMD

All the selected males were separately examined in terms of the translated version of Diagnostic Criteria for TMD (DC/TMD Axis I) by two calibrated examiners. The patients received one or more of the following diagnoses: muscle disorders (group I), disc displacement (group II), or arthralgia, osteoarthritis, or osteoarthrosis (group III). The examiners had passed a specific DC/TMD training course on the management of TMD in their 2‐year fellowship program. Optimal interobserver reliability was ensured before the study (the kappa value was 0.90).

### 2.6 Statistical methods

The chi‐square and Mann–Whitney tests were applied to evaluate the relationship between the level of depression and TMD. The level of significance was set at *p* < .05. The chi‐square test was applied to assess the presence of any relationship between depression and clinical symptoms of TMD. The Mann–Whitney test was applied to evaluate the relationship between the level of depression and clinical symptoms of TMD. IBM‐SPSS statistical software version 22 was used for analyzing the data.

## 3 RESULTS

**Figure 1:**
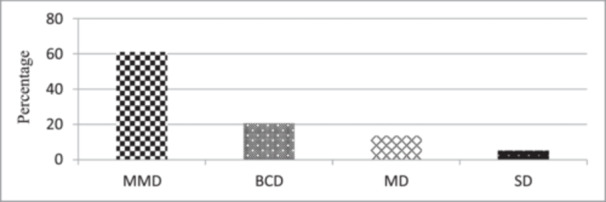
Frequency of each type of depression based on Beck's Depression Inventory. BCD, borderline clinical depression; MD, moderate depression; MMD, mild mood disturbance; SD, severe depression.

A total of 144 individuals with different levels of depression met the inclusion criteria. All of them were male conscripts with a mean age of 20.7 ± 1.92 years. The control group consisted of 138 young males without chronic stress with a mean age of 21.1 ± 1.99 years. The two groups had no significant difference regarding the mean age (*p* = .21). The total number of participants diagnosed with mild mood disturbance was the highest followed by borderline clinical, moderate, and server depression. There was no participant with extreme depression in the young conscripts (Table 3 and Figure 1).

**Table 3:** Demographic and psychiatric data of the study population.

Age		Level of depression	
Range	*n* (percentage)	Characteristics	*n* (percentage)
18–19	32 (22.2)	BCD	30 (20.8)
19–20	30 (20.8)	MMD	87 (60.4)
20–21	24 (16.6)	BCD	30 (20.8)
21–22	18 (12.8)	MD	19 (13.19)
22–23	14 (9.7)	SD	8 (5.55)
23–24	16 (11.1)	ED	0 (0)
24–25	10 (6.9)		

Abbreviations: BCD, borderline clinical depression; ED, extreme depression; MD, moderate depression; MMD, mild mood disturbance; SD, severe depression.

**Figure 2:**
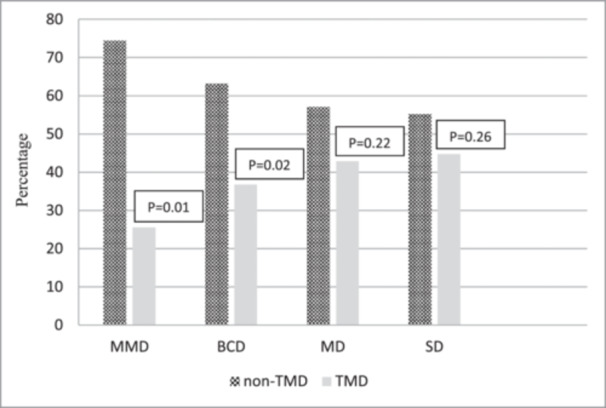
Frequency of TMD in each level of depression based on Beck's Depression Inventory. BCD, borderline clinical depression; MD, moderate depression; MMD, mild mood disturbance; non‐TMD, without temporomandibuar disorders; SD, severe depression; TMD, temporomandibuar disorders.

TMD evaluation among depressed participants showed that the majority of male conscripts (67.3%) had more than one TMD diagnosis (Table 4). The combination of groups I, II, and III had the highest frequency among the depressed groups (*p* = .03). Muscle disorders alone (group I) were detected in 5.1% of them; however, groups II and III diagnoses alone were noted in 12.3% and 16.4% of patients, respectively. It was noted that 21% of the control males showed at least one of the TMD diagnoses; however, this rate was 100% for patients with severe depression (Figure 2).

**Table 4:** Frequency of different DC/TMD diagnoses in each group.

Level of depression	DC/TMD groups							Total (*N*)
	I (%)	II (%)	III (%)	I+II (%)	I+III (%)	II+III (%)	I+II+III (%)	
MMD	1 (2.2)*	6 (13.6)	8 (18.1)	0 (0)*	7 (15.9)	9 (20.4)	13 (29.5)^a^	44
BCD	2 (7.1)	4 (14.2)	4 (14.2)	2 (7.14)	5 (17.8)	6 (21.4)^a^	5 (17.8)	28
MD	1 (5.8)	2 (11.7)	2 (11.7)	1 (5.8)	3 (17.6)	5 (29.4)^a^	3 (17.6)	17
SD	1 (12.5)	0 (0)*	2 (25)	0 (0)*	1 (12.5)	1 (12.5)	3 (37.5)^a^	8
Control	4 (13.7)	13 (44.8)*	2 (6.8)	6 (20.6)	1 (3.4)	3 (10.3)	0 (0)*	29

Abbreviations: BCD, borderline clinical depression; MD, moderate depression; MMD, mild mood disturbance; SD, severe depression.

^a^ *p* < .05 compared with other groups.

* *p* < .01 compared with other groups.

A combination of group I, II, and III was the most frequent diagnosis in patients with mild mood disturbance (29.5%) and severe depression (37.5%). However, in patients with borderline clinical and moderate depression, the combination of group I and II was the most frequent DC/TMD diagnoses. In the control population, group II were more frequent than other groups (44.8%); however, a combination of group I and III disorders had the least frequency (3.4%).

The obtained data revealed that patients with borderline clinical depression, moderate depression, and severe depression were significantly more likely to have one of the DC/TMD diagnoses (*p* = .03, *p* = .02, *p* = .02). However, no significant difference was noted among them regarding this respect (*p* = .22).

## 4 DISCUSSION

In this study, the relationship between different levels of depression and TMD was investigated in Iranian male conscripts with chronic stress. Selection of this particular population was because the authors believe that this population is more susceptible to chronic stress. Previous studies have shown a direct relationship between age and sex and TMD. Also, it has been reported that females are more likely to have TMD symptoms (Bitiniene et al., 2018). Unlike previous studies, young males were selected as the target population of the present study because conscription is a mandatory military service for young males in Iran, while females do not have such an experience.

The most frequent diagnosis which was based on the DC/TMD criteria was different for each level of depression; however, the pain experience was common among the Iranian population, which was either reported alone or in combination with other symptoms. Another study reported similar findings in a depressed Chinese population (Liao et al., 2011). They reported pain in the masticatory muscles or TMJ as the most prevalent TMD symptom. In the control group, disc displacement was the most frequent DC/TMD diagnosis, which was different from the diagnoses for the depressed groups. This difference could be attributed to the effect of chronic stress on TMD symptoms.

It was reported that “it is not easy to find the priority when considering TMD and depression” (Slade et al., 2007). In other words, the authors could not find out whether depression resulted in TMD or vice versa. The results of the present study on the Iranian males revealed a direct relationship between the level of depression and percentage of participants with TMD symptoms. Another study conducted in Singapore reported that patients with TMD were at higher risk of depression. This association might be attributed to myofascial and preauricular pain occurrence in the affected individuals (Yap et al., 2003). Such chronic pain could increase the patients’ stress and anxiety level and subsequently lead to depression. The association of stress and masticatory movement disorders could also explain some parts of the results of the present study. Poveda Poveda Roda et al. demonstrated that stressed individuals experience more parafunctional habits (Poveda Roda et al., 2007). They also reported a significant association between the masticatory movement disorders and TMD. A recent systematic review reported similar results, and the authors found a significant correlation between different levels of stress and depression (Madsen et al., 2017). Such results could explain the findings of the present study.

Young men experience tough days in the mandatory military service (conscription), especially in the first 6 months of serving. Therefore, many of them may be prone to chronic stress, which may enhance their susceptibility to depression and TMD at the same time. Minghelli et al. demonstrated a significant association between the level of anxiety and depression in a Portuguese population. Although the participants of their study were different from those of the present study (college students with high stress level were selected as the target population in their study), their findings were in line with those of the current study. Moreover, another study on a Turkish population reported a significant association between TMD and psychological disorders such as depression and anxiety (Diraçoglu et al., 2016). Clinically, the results of the present study could be alarming for practitioners who encounter or treat male conscripts or patients under continuous stress.

This study had some intrinsic limitations including selective participants (only males). Therefore, the results of this study may not be generalized to the female population who are at high risk of TMD. As well, the application of clinical based methods for TMD evaluation without radiographic intervention (according to the DC/TMD guidelines) might miss some patients. Due to the abovementioned limitations, results of these local cross‐sectional studies should generalize with caution. Further international and multiethnic studies with a larger population could decrease the limitations and reveal more accurate information about this topic.

## 5 CONCLUSION

It could be concluded that the level of depression is associated with the TMD in young males under chronic stress. The incidence of TMD increased by an increase in the severity of depression. This population showed higher incidence of TMD compared with peers without chronic stress. The percentage of individuals diagnosed with TMD was the highest in the severe depression group followed by moderate depression, borderline clinical depression, mild mood disturbance, and the control groups.

## AUTHOR CONTRIBUTIONS

All authors have contributed to this study and the paper in equal parts. Behzad Salari and Maryam Sadrzadeh Afshar had the idea for the research, participated in the design of the study, and literature research, wrote the manuscript, and carried out proofreading. Sina Varasteh Nejad participated in the design of the study, interpreted data, and performed the statistical analysis. All authors agree with the content of the manuscript.

## CONFLICT OF INTEREST STATEMENT

The authors declare no conflict of interest.

## Data Availability

The data that support the findings of this study are available on request from the corresponding author. The data are not publicly available due to privacy or ethical restrictions.
